# Maternal multimorbidity and preterm birth in Scotland: an observational record-linkage study

**DOI:** 10.1186/s12916-023-03058-4

**Published:** 2023-09-12

**Authors:** Amaya Azcoaga-Lorenzo, Adeniyi Francis Fagbamigbe, Utkarsh Agrawal, Mairead Black, Muhammad Usman, Siang Ing Lee, Kelly-Ann Eastwood, Ngawai Moss, Rachel Plachcinski, Catherine Nelson-Piercy, Sinead Brophy, Dermot O’Reilly, Krishnarajah Nirantharakumar, Colin McCowan, on behalf of the MuM-PreDiCT Group

**Affiliations:** 1 Division of Population and Behavioural Sciences, School of Medicine, University of St Andrews, St Andrews, UK; 2 Hospital Rey Juan Carlos, Instituto de Investigación Sanitaria Fundación Jimenez Diaz, Madrid, Spain; 3 Research Network On Chronicity, Primary Care and Prevention and Health Promotion, (ISCIII), Madrid, Spain; 4 Department of Epidemiology and Medical Statistics, College of Medicine, University of Ibadan, Ibadan, Nigeria; 5 Institute of Applied Health Sciences, School of Medicine, Medical Sciences & Nutrition, University of Aberdeen, Aberdeen, UK; 6 Nuffield Department of Primary Care Health Sciences, University of Oxford, Oxford, UK; 7 Aberdeen Centre for Women’s Health Research, School of Medicine, Medical Science and Nutrition, University of Aberdeen, Aberdeen, UK; 8 Institute of Applied Health Research, University of Birmingham, Birmingham, UK; 9 Centre for Public Health, Queen’s University of Belfast, Belfast, UK; 10 St Michael’s Hospital, University Hospitals Bristol and Weston NHS Foundation Trust, Bristol, UK; 11 Patient and Public Representative, London, UK; 12 Guy’s and St. Thomas’ NHS Foundation Trust, London, UK; 13 Data Science, Medical School, Swansea University, Swansea, UK

**Keywords:** Multimorbidity, Pregnancy, Premature birth, Electronic health records, Generalised Estimating Equation

## Abstract

**Background:**

Multimorbidity is common in women across the life course. Preterm birth is the single biggest cause of neonatal mortality and morbidity. We aim to estimate the prevalence of multimorbidity in pregnant women and to examine the association between maternal multimorbidity and PTB.

**Methods:**

This is a retrospective cohort study using electronic health records from the Scottish Morbidity Records. All pregnancies among women aged 15 to 49 with a conception date between 1 January 2014 and 31 December 2018 were included. Multimorbidity was defined as the presence of two or more pre-existing long-term physical or mental health conditions, and complex multimorbidity as the presence of four or more. It was calculated at the time of conception using a predefined list of 79 conditions published by the MuM-PreDiCT consortium. PTB was defined as babies born alive between 24 and less than 37 completed weeks of gestation. We used Generalised Estimating Equations adjusted for maternal age, socioeconomic status, number of previous pregnancies, BMI, and smoking history to estimate the effect of maternal pre-existing multimorbidity. Absolut rates are reported in the results and tables, whilst Odds Ratios (ORs) are adjusted (aOR).

**Results:**

Thirty thousand five hundred fifty-seven singleton births from 27,711 pregnant women were included in the analysis. The prevalence of pre-existing multimorbidity and complex multimorbidity was 16.8% (95% CI: 16.4–17.2) and 3.6% (95% CI: 3.3–3.8), respectively. The prevalence of multimorbidity in the youngest age group was 10.2%(95% CI: 8.8–11.6), while in those 40 to 44, it was 21.4% (95% CI: 18.4–24.4), and in the 45 to 49 age group, it was 20% (95% CI: 8.9–31.1). In women without multimorbidity, the prevalence of PTB was 6.7%; it was 11.6% in women with multimorbidity and 15.6% in women with complex multimorbidity. After adjusting for maternal age, socioeconomic status, number of previous pregnancies, Body Mass Index (BMI), and smoking, multimorbidity was associated with higher odds of PTB (aOR = 1.64, 95% CI: 1.48–1.82).

**Conclusions:**

Multimorbidity at the time of conception was present in one in six women and was associated with an increased risk of preterm birth. Multimorbidity presents a significant health burden to women and their offspring. Routine and comprehensive evaluation of women with multimorbidity before and during pregnancy is urgently needed.

**Supplementary Information:**

The online version contains supplementary material available at 10.1186/s12916-023-03058-4.

## Background

Multimorbidity, defined as the presence of two or more long-term physical or mental health conditions, is common in women and strongly associated with age and socioeconomic deprivation in the general population [1] Recent studies have shown that while multimorbidity risk increases with age [2], this relationship is not linear and may vary across different age groups [3]. A previous study on data from 2018 reported that one in five pregnant women and birthing people were living with multimorbidity in the UK, highlighting the potential for complications in pregnancy and implications for the offspring [4]. However, the impact of multimorbidity on maternal and pregnancy outcomes is poorly understood as most research has focused on individual maternal diseases to date. There is limited knowledge on the prevalence [5, 6], causes and consequences of multimorbidity during pregnancy on maternal and birth, and neonatal outcomes [7–10]. One recent paper has reported an association between maternal multimorbidity and preterm birth (PTB) and other adverse perinatal outcomes [10]

PTB is defined as babies born alive before 37 weeks of pregnancy [11, 12]. It is a significant concern globally, as it is one of the leading causes of perinatal and neonatal morbidity and mortality. While developed countries generally have higher survival rates for preterm babies, they still face an elevated risk of respiratory and gastrointestinal complications, severe infections, and neurodevelopmental impairments [11, 13]. Prematurity remains the primary cause of death among children under the age of 5 years worldwide [14, 15]. Statistics reveal that the PTB rate in the UK has remained unchanged for the past decade. In 2012, over 52,000 babies in England and Wales were born prematurely, accounting for around 7.3% of live births, while Scotland experienced its highest recorded rate of 6.8% for live singleton births in 2018/19 [14]. Despite advances in understanding risk factors and mechanisms associated with PTB, along with the implementation of various public health and medical interventions aimed at its reduction, the problem persists.

Although associations between PTB and a wide range of socio-demographic, medical, obstetric, fetal, and environmental factors have been reported, approximately two-thirds of PTBs occur without an evident risk factor [16] In this context, assessing the possible association between birth outcomes and multimorbidity during pregnancy will contribute to knowledge on how the presence of multiple long-term conditions affects women and their children and potentially contribute to the development of specific interventions to reduce the burden of PTB.

This study aimed to estimate the prevalence of multimorbidity in pregnant women in Scotland between 2014 and 2018 and examine the association between maternal multimorbidity and PTB. As a secondary objective, we explored the association of maternal multimorbidity with stillbirth, neonatal admission, and neonatal death.

## Methods

### Study design, data source, and linkage

This is a retrospective cohort study using electronic health records (EHRs) from two Scottish regional health boards: National Health Service (NHS) Tayside and NHS Fife, provided by The Health Informatics Centre (HIC) based at the University of Dundee which represents approximately 20% of the Scottish population [17]. The dataset was created using the Scottish Morbidity Records (SMR) [18] (Additional file 1: Data source) by linking Scottish Maternity Records (SMR02) to data from Hospital Admissions (SMR01), Mental Health Inpatients (SMR04), Accident and Emergency, demography records and National Records of Scotland Death registration. This covered diagnoses and demographic data for all inpatient stays and day cases in hospital for residents in the two regions. The dataset was also linked to the Prescribing Information System for data on all medications from primary care dispensed in the community. Pregnancies were identified from maternity records (SMR02). Deterministic data linkage of different databases is carried out using the community health index number–a unique identifier for each patient– used in all healthcare contacts across Scotland. Access to the datasets by the authorised researchers was exclusively within a Safe Haven environment after deidentification of the records.

### Study population

We included all singleton pregnancies among women aged 15 to 49 in Tayside and Fife, where the estimated conception date was between 1 January 2014 to 31 December 2018, and women had been a resident in the area for at least one year prior to the conception date. Two different subsets of the study population were used to address the study’s objectives. To estimate the prevalence of multimorbidity and its associated characteristics, all pregnancies in the 5 year period were included. In cases where an individual woman had more than one pregnancy during the period of interest, one pregnancy was randomly selected and considered the index pregnancy for the prevalence analysis. To investigate the association of multimorbidity with PTB, all pregnancies within the 5 years were analysed, and pregnancies from the same women were treated as related records.

### Maternal baseline information: covariates

We identified characteristics of interest at the estimated date of conception for each pregnancy, namely age, number of previous live births and pregnancies, smoking history, nutritional status according to BMI at the start of the pregnancy, ethnicity, and socioeconomic status measured by quintiles of the Scottish Index of Multiple Deprivation (SIMD). The SIMD is the standard measure of socio-economic deprivation at the small area level (data zones) used in policy and research in Scotland. It includes measures relating to income, employment, education, health, access to services, crime, and housing [19].

**Fig. 1:**
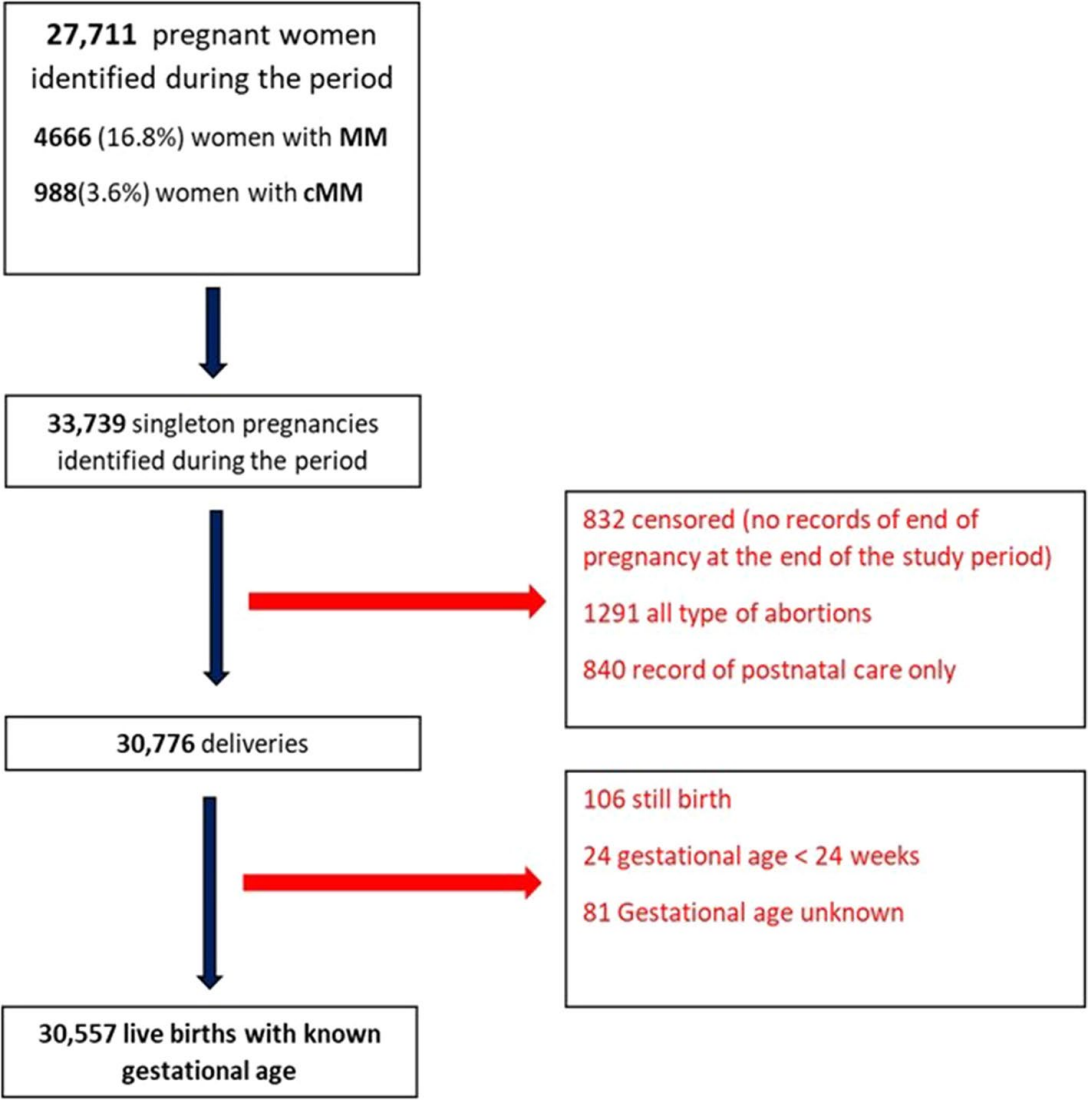
Flow chart for selection of study population

The date of conception was based on the last menstrual period (LMP) recorded at booking appointment or estimated by considering the difference between the recorded gestational age at delivery or booking appointment if the former was unavailable. Gestational age at birth was established based on the gestational age recorded at delivery. Women whose data did not meet standard quality checks were excluded (Fig. 1: Flow chart and Additional file 2: Cohort selection).

### Multimorbidity definition: outcome and exposure

Multimorbidity was defined by the presence of two or more pre-existing long-term physical or mental health conditions. We also defined complex multimorbidity as the presence of four or more of these conditions [20]. Multimorbidity was calculated at the estimated time of conception for each pregnancy using a predefined list of 79 conditions published by the MuM-PreDiCT consortium [4]. The list of conditions was selected by a multidisciplinary group and patient representatives for pregnant women. Identification of these 79 long-term health conditions was determined using a combination of codes from the International Classification of Disease 10th version (ICD-10) and defined prescribing indicators from the Community Prescribing Information System. The definition has demonstrated reproducibility across different data sets. The exact multimorbidity definition was used for evaluating the prevalence of multimorbidity and as an exposure variable for the models examining PTB and other outcomes. A complete list of the health conditions and codes is provided in Additional file 3: Phenome definition.

### Outcomes

Preterm birth was defined as babies born alive between 24 and 37 completed weeks of pregnancy. According to the World Health Organisation [15] definitions, we established sub-categories of preterm birth, based on gestational age at birth as follows: extremely preterm (less than 28 weeks), very preterm (28 to less than 32 weeks) and moderate to late preterm (32 to less than 37) weeks. We established the lower limit at 24 weeks in line with current clinical practice and to be consistent with the definitions used in our databases [21, 22]. The number of births between 22 and < 24 weeks recorded as live births has been reported.

Neonatal death was defined as a livebirth dying, and neonatal admissions as any admission of any length to a neonatal unit after delivery but before the 28th day.

Variable definitions in our study are based on the available information in the original datasets. A detailed definition of each variable and how they are recorded in the datasets is available in Additional file 4: Outcome and variable definition.

### Statistical analysis

We summarised the characteristics of all women that met the inclusion criteria and their pregnancy outcomes using counts and proportions. The characteristics were also reported by the sub-categories of preterm birth. To estimate the prevalence of multimorbidity, women were included only once by a random selection of one of their pregnancies if they had more than one during the period. We included all pregnancies to estimate the effect of maternal pre-existing multimorbidity on preterm birth, stillbirth, neonatal admission, and neonatal death. We used Generalised Estimating Equation to account for one woman having more than one pregnancy during the study period. The model was adjusted for maternal age, socioeconomic status, ethnicity, number of previous pregnancies, BMI, and smoking history. Multimorbidity was re-calculated for each pregnancy for women having more than one pregnancy within the study period. We conducted a stratified analysis with a regression model using the Mantel–Haenszel approach. Also, we investigated the potential interaction effect between multimorbidity and age and deprivation in the likelihood of preterm birth. Rates (proportions) in the results and tables are absolute, whereas the ORs given are adjusted (aOR). Analysis was carried out using R (v3.6.3) and the Stata statistical software package (version 14). Missing data were assigned to separate categories, reported in the descriptive statistics, and accounted for in the regression models, so all women were entered into the model. The study is reported in accordance with the RECORD guideline provided in Additional file 5: STROBE check list.

## Results

Overall, 27,771 pregnant women aged 15–49 years were included in the analysis to estimate the prevalence of multimorbidity during the 5-year period (Fig. 1.)

The characteristics of the cohort are presented in Table 1. Most of the women were 20–34 years old (76.8%), of white ethnicity (75%), and 41.2% were in the most deprived categories (SIMD 1 and 2). A majority had a normal weight according to BMI (35.8%), although 21.7% were overweight and 19.4% obese. The prevalence of pre-existing multimorbidity and complex multimorbidity in pregnant women was 16.8% (95% CI: 16.4–17.2) and 3.6% (95% CI: 3.3–3.8), respectively. The prevalence of multimorbidity in the youngest age group was 10.2% (95% CI: 8.8–11.6), while in the 40 to 44 age group, it was 21.4% (95% CI: 18.4–24.4), and in the 45 to 49 age group, it was 20% (95% CI:8.9–31.1). Among women with no previous pregnancies, 13.6% (95% CI:12.9–14.3) had multimorbidity, whereas, among those with six or more previous pregnancies, the prevalence was 34.9% (95% CI: 31.3–38.5). Multimorbidity was prevalent in 20.6% (95% CI:19.6–21.7) of women living in the most deprived areas, whereas among those residing in the least deprived areas, it was 12.1% (95% CI:11–13.2).

**Table 1:** Sociodemographic characteristics and prevalence of multimorbidity and complex multimorbidity of pregnant women

	**Total cohort** n (column %)	**Non-MM n [row % (CI)]**	**MM (2 +) n [row % (CI)]**	**cMM (4 +) n [row % (CI)]**
Total	27771(100)	23105 [83.2(82.8–83.6)]	4666 [16.8(16.4–17.2)]	988 [3.6(3.3–3.8)]
**Age at conception (years)**				
15–19	1779(6.4)	1597 [89.8(88.4–91.2)]	182 [10.2(8.8–11.6)]	21 [1.2(0.7–1.7)]
20–24	5048(18.2)	4161 [82.4(81.4–83.5)]	887 [17.6(16.5–18.6)]	160 [3.2(2.7–3.7)]
25–29	8288(29.8)	6877[83.0(82.2–83.8)]	1411 [17(16.2–17.8)]	275 [3.3(2.9–3.7)]
30–34	8000(28.8)	6699[83.7(82.9–84.5)]	1301[16.3(15.5–17.1)]	289 [3.6(3.2–4.0)]
35–39	3881(14)	3161[81.4(80.2–82.7)]	720[18.6(17.3–19.8)]	208 [5.4(4.7–6.1)]
40–44	725(2.6)	570[78.6(75.6–81.6)]	155[21.4(18.4–24.4)]	34 [4.7(3.2–6.2)]
45–49	50(0.2)	40[80.0(68.9–91.1)]	10[20.0(8.9–31.1)]	< 5
**Previous live birth**				
0	11269 (40.6)	9668 [85.8(85.1–86.4)]	1601[14.2(13.6–14.9)]	300 [2.7(2.4–3.0)]
1	10091 (36.3)	8538[84.6(83.9–85.3)]	1553 [15.4(14.7–16.1)]	330 [3.3(2.9–3.6)]
2	3693 (13.3)	2863 [77.5(76.2–78.9)]	830 [22.5(21.1–23.8)]	184 [5.0(4.3–5.7)]
3	1382 (5)	1048 [75.8(73.6–78.1)]	334 [24.2(21.9–26.4)]	94 [6.8(5.5–8.1)]
4	403 (1.5)	287 [71.2(66.8–75.6)]	116 [28.8(24.4–33.2)]	28 [6.9(4.5–9.4)]
5 +	272 (1)	192 [70.6(65.2–76.0)]	80.0 [29.4(24.0–34.8)]	16 [5.9(3.1–8.7)]
Missing	661 (2.4)	509 [77.0(73.8–80.2)]	152 [23.0(19.8–26.2)]	36 [5.4(3.7–7.2)]
**Previous pregnancies**				
0	8531(30.7)	7370 [86.4(85.7–87.1)]	1161[13.6(12.9–14.3)]	210 [2.5(2.1–2.8)]
1	8926 (32.1)	7704 [86.3(85.6–87)]	1222 [13.7(13.0–14.4)]	236 [2.6(2.3–3.0)]
2	4956 (17.9)	4068 [82.1(81–83.2)]	888 [17.9(16.8–19.0)]	193 [3.9(3.4–4.4)]
3	2622 (9.4)	2049[78.1(76.6–79.7)]	573 [21.9(20.3–23.4)]	134 [5.1(4.3–6.0)]
4	1329 (4.8)	973 [73.2(70.8–75.6)]	356 [26.8(24.4–29.2)]	94 [7.1(5.7–8.5)]
5	701(2.5)	477 [68.0(64.6–71.5)]	224 [32.0(28.5–35.4)]	51 [7.3(5.4–9.2)]
6 +	685 (2.5)	446 [65.1(61.5–68.7)]	239 [34.9(31.3–38.5)]	70 [10.2(8.0–12.5)]
Missing	21 (0.1)	18 [85.7(70.7–100.7)]	< 5	< 5
**Ethnicity**				
White	20836 (75)	17375 [83.4(82.9–83.9)]	3461 [16.6(16.1–17.1)]	722 [3.5(3.2–3.7)]
Mixed race	43 (0.2)	39 [90.7(82–99.4)]	< 5	< 5
Black	140 (0.5)	131 [93.6(89.5–97.6)]	9 [6.4(2.4–10.5)]	< 5
Asian	570 (2.1)	529 [92.8(90.7–94.9)]	41 [7.2(5.1–9.3)]	8 [1.4(0.4–2.4)]
Others	411 (1.5)	343[83.5(79.9–87)]	68 [16.5(13–20.1)]	17 [4.1(2.2–6.1)]
Missing	5771(20.8)	4688 [81.2(80.2–82.2)]	1083 [18.8(17.8–19.8)]	240 [4.2(3.6–4.7)]
**Deprivation (SIMD)**				
Most deprived	6056 (21.8)	4806 [79.4(78.3–80.4)]	1250 [20.6(19.6–21.7)]	293 [4.8(4.3–5.4)]
2	5382 (19.4)	4318 [80.2(79.2–81.3)]	1064 [19.8(18.7–20.8)]	259 [4.8(4.2–5.4)]
3	4653 (16.8)	3894 [83.7(82.6–84.7)]	759[16.3(15.3–17.4)]	150 [3.2(2.7–3.7)]
4	4883 (17.6)	4248 [87(86.1–87.9)]	635 [13(12.1–13.9)]	119 [2.4(2–2.9)]
Least Deprived	3441(12.4)	3024 [87.9(86.8–89)]	417 [12.1(11–13.2)]	69 [2(1.5–2.5)]
Missing	3356 (12.1)	2815 [83.9(82.6–85.1)]	541 [16.1(14.9–17.4)]	98 [2.9(2.4–3.5)]
**BMI**				
Underweight (< 18.5)	583 (2.1)	500 [85.8(82.9–88.6)]	83 [14.2(11.4–17.1)]	11 [1.9(0.8–3.0)]
Normal Weight (18.5–24.9)	9940 (35.8)	8603 [86.5(85.9–87.2)]	1337 [13.5(12.8–14.1)]	250 [2.5(2.2–2.8)]
Overweight (25–29.9)	6036 (21.7)	5117 [84.8(83.9–85.7)]	919 [15.2(14.3–16.1)]	173 [2.9(2.4–3.3)]
Obese (> 30)	5383 (19.4)	4262 [79.2(78.1–80.3)]	1121 [20.8(19.7–21.9)]	259 [4.8(4.2–5.4)]
Missing	5829 (21)	4623 [79.3(78.3–80.4)]	1206 [20.7(19.6–21.7)]	295 [5.1(4.5–5.6)]
**Smoking History**				
Never smoked	16619 (59.8)	14429 [86.8(86.3–87.3)]	2190 [13.2(12.7–13.7)]	397 [2.4(2.2–2.6)]
Current smoker	5373 (19.4)	3956 [73.6(72.4–74.8)]	1417 [26.4(25.2–27.6)]	356 [6.6(6.0–7.3)]
Former smoker	3579 (12.9)	2912 [81.4(80.1–82.6)]	667 [18.6(17.4–19.9)]	139 [3.9(3.3–4.5)]
Not known	2200 (7.9)	1808 [82.2(80.6–83.8)]	392 [17.8(16.2–19.4)]	96 [4.4(3.5–5.2)]

*MM* Multimorbidity, *cMM* Complex multimorbidity, *SIMD* Scottish Index of Multiple Deprivation, *BMI* Body Mass Index

**Fig. 2:**
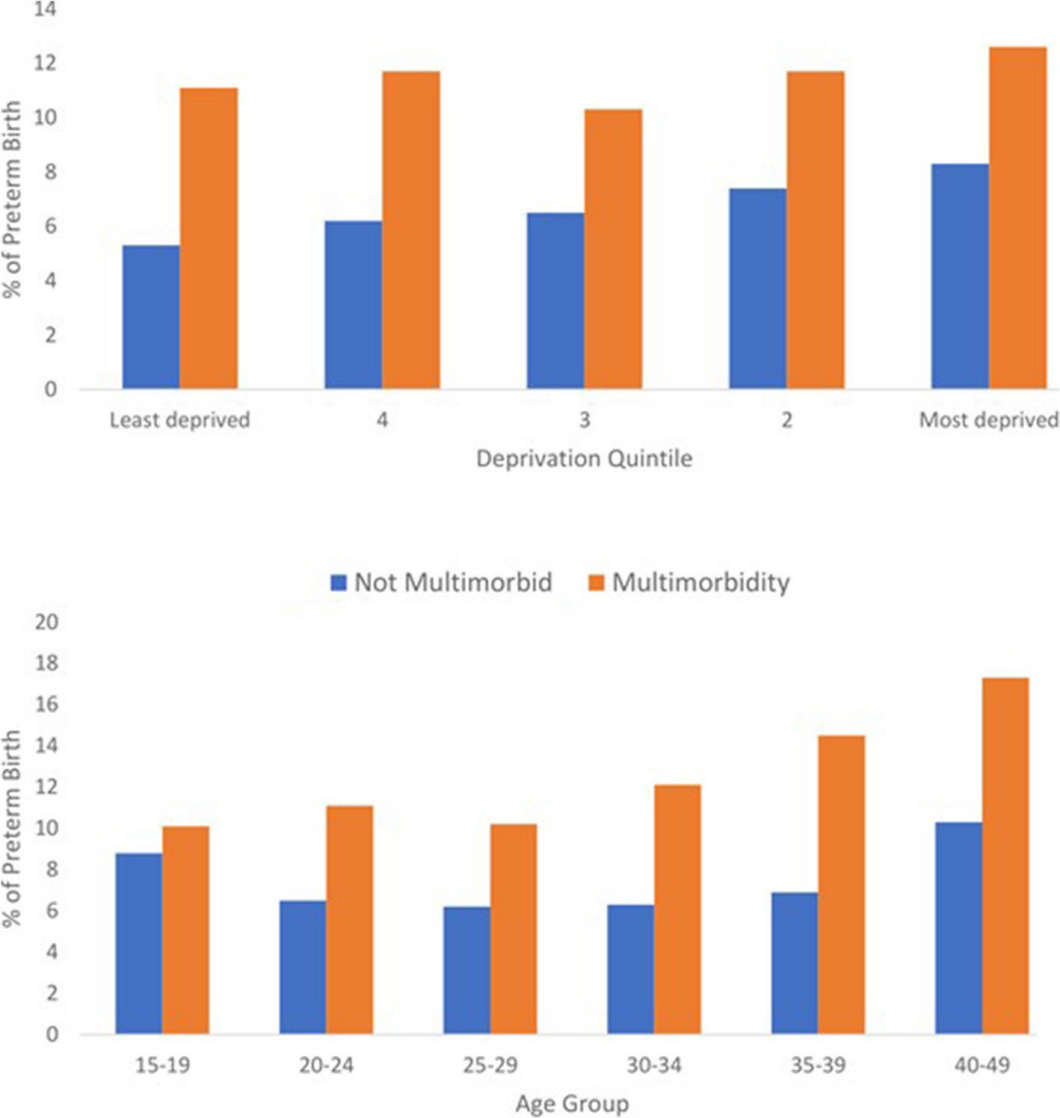
Prevalence of PTB by multimorbidity, age, and deprivation

During the study period, we identified 30,557 live births with known gestational age from women who gave birth ≥ 24 weeks of gestation. The prevalence of overall preterm birth was 7.5%. In women with no identified multimorbidity, the prevalence of PTB was 6.7%, while it was 11.6% in women with multimorbidity and 15.6% in women with complex multimorbidity. Vaginal birth was the mode of delivery in 60.7% of women without chronic conditions, 57% of women with maternal multimorbidity, and 53.1% of women with complex multimorbidity. Among women with multimorbidity, 16.5% had an emergency c-section, while 17.2% had an elective c-section, as opposed to 14.9% and 12.9%, respectively, among women without multimorbidity. Neonatal admissions were reported in 5.6% (1426) of births from women without multimorbidity, 9.3% (471) from women with multimorbidity, and 14.3% (149) from women with complex multimorbidity (Table 2). The prevalence of PTB by age, deprivation, and multimorbidity are presented in Figs. 1 and 2.

**Table 2:** Perinatal characteristics of singleton births among women with and without multimorbidity

	**Total number of single births n (%)**	**Births in women with no MM pre-pregnancy n (%)**	**Births in women with MM: 2 +) pre- pregnancy n(%)**	**Births in women with complex MM: 4 +) pre-pregnancy n (%)**
Livebirth (with known gestational age)	30557	25486	5071	1041
Term	28260 (92.5)	23783 (93.3)	4477 (88.3)	879 (84.4)
**Preterm births (< 37 weeks of gestation)**	2297 (7.5)	1703 (6.7)	594 (11.6)	162 (15.6)
Moderate preterm (32 to < 37 completed weeks of gestation)	1970 (5.8)	1451 (5.7)	519 (10.2)	139 (13.4)
Very preterm (28 to < 32 weeks completed weeks of gestation)	238 (0.8)	181 (0.7)	57 (1.1)	18 (1.7)
Extremely preterm (24 to < 28 weeks completed weeks of gestation)	89 (0.3)	71 (0.3)	18 (0.4)	5(0.5)
**Birth weight**				
Normal (> = 2500 gr)	27926 (92.7)	23436 (93.4)	4490 (89.5)	879 (84.7)
Low Birth Weight (1500 to 2499 gr)	1647(5.5)	1235(4.9)	412 (8.2)	118 (11.4)
Very Low Birth Weight (1000 to 1499 gr)	184 (0.6)	133(0.5)	51(1.0)	16(1.5)
Extremely Low Birth Weight (< 1000gr)	98 (0.3)	80(0.3)	18 (0.4)	8 (0.8)
Missing	702 (2.3)	602(2.4)	100 (2)	20 (1.9)
**Mode of birth**				
Vaginal	18506 (60.7)	15618 (61.3)	2888 (57)	553 (53.1)
Instrumental	2619 (8.6)	2243 (8.8)	376 (7.4)	74 (7.1)
Breech	94 (0.3)	74 (0.3)	20 (0.4)	< 5(0.4)
Elective C-section	4095 (13.4)	3231 (12.7)	864 (17)	203 (19.5)
Emergency C-section	4539 (14.9)	3713 (14.6)	826 (16.3)	187(18)
Missing	704 (2.3)	607 (2.4)	97 (1.9)	20 (1.9)
**Stillbirth**	106 (0.3)	90 (0.4)	16 (0.3)	6 (0.6)
**Neonatal admission**	1897 (5.6)	1426 (5.6)	471(9.3)	149 (14.3)
**Neonatal deaths**	74 (0.2)	48(0.2)	26 (0.5)	10 (1.0)

After adjusting for maternal age, socioeconomic status, smoking history, BMI, and number of previous pregnancies, multimorbidity was associated with higher odds of PTB (aOR = 1.64, 95% CI: 1.48–1.82). Maternal multimorbidity was also significantly associated with moderate PTB (aOR = 1.69, 95% CI: 1.52–1.89), but it was not significantly associated with very PTB (aOR = 1.22, 95% CI: 0.9–1.66) and extremely PTB (aOR = 0.86, 95% CI: 0.51–1.44) (Table 3). Women with multimorbidity had a higher risk of PTB compared to women without multimorbidity, except for those in the youngest age group (15 to 19 years), and across various socioeconomic levels (Additional file 6: Table 4). Given that age and higher levels of deprivation are associated with multimorbidity, we examined potential interaction effects to investigate the relationship between age, levels of deprivation, and the odds of PTB. However, our analysis did not reveal any significant interaction. Consequently, the impact of multimorbidity on the odds of PTB did not significantly differ across different age groups or levels of deprivation.

**Table 3:** Multivariate adjusted association between maternal multimorbidity and preterm birth

	**Preterm birth** **(24 to < 37 w)** Adjusted OR (95% CI)	**Moderate preterm** **(32 to < 37 w)** Adjusted OR (95% CI)	**Very preterm** **(28 to < 32 w)** Adjusted OR (95% CI)	**Extremely preterm** **(24 to < 28 w)** Adjusted OR (95% CI)
**Maternal multimorbidity**	**1.64 (1.48–1.82)**	**1.69 (1.52–1.89)**	**1.22 (0.9–1.66)**	**0.86 (0.51–1.44)**
**No MM**	**Ref**	**Ref**	**Ref**	**Ref**
Adjusted for maternal age, socioeconomic status, ethnicity, number of previous pregnancies, BMI and smoking history. SIMD: Scottish Index of Multiple Deprivation. BMI: body mass index				
**Age at conception, years**				
15–19	1.13 (0.94–1.36)	1.13 (0.92–1.38)	1 (0.56–1.78)	1.79 (0.87–3.7)
20–24	1 (0.87–1.14)	0.97 (0.85–1.12)	1.22 (0.84–1.79)	1.37 (0.79–2.38)
25–29	Ref	Ref	Ref	Ref
30–34	1.11 (0.99–1.25)	1.06 (0.94–1.2)	1.48 (1.05–2.09)	1.09 (0.64–1.86)
35–39	1.24 (1.08–1.44)	1.18 (1.01–1.37)	1.49 (0.97–2.27)	1.18 (0.61–2.26)
40–44	1.72 (1.35–2.21)	1.7 (1.32–2.2)	1.14 (0.48–2.71)	0.73 (0.17–3.21)
45–49	1.07 (0.33–3.46)	1.03 (0.32–3.31)	ISS	ISS
**SIMD**				
Most deprived 1	1.31 (1.1–1.55)	1.18 (0.99–1.41)	3.34 (1.73–6.46)	1.11 (0.53–2.34)
2	1.27 (1.07–1.51)	1.16 (0.97–1.39)	3.28 (1.69–6.35)	1.25 (0.59–2.63)
3	1.14 (0.95–1.36)	1.06 (0.88–1.28)	2.34 (1.18–4.67)	1.11 (0.51–2.41)
4	1.11 (0.93–1.32)	1.03 (0.86–1.23)	2.3 (1.16–4.56)	0.97 (0.44–2.14)
Least deprived 5	Ref	Ref	Ref	Ref
Missing	1.04 (0.85–1.26)	1 (0.81–1.22)	2.19 (1.06–4.53)	0.61 (0.23–1.62)
**Previous pregnancies**				
0	Ref	Ref	Ref	Ref
1	0.76 (0.68–0.85)	0.8 (0.71–0.9)	0.57 (0.4–0.81)	0.67 (0.41–1.12)
2	0.8 (0.7–0.91)	0.81 (0.7–0.94)	0.68 (0.46–1.02)	0.75 (0.41–1.36)
3	0.92 (0.79–1.08)	0.9 (0.76–1.07)	1.13 (0.74–1.73)	0.92 (0.45–1.86)
4	1.03 (0.84–1.25)	1.01 (0.82–1.25)	1.11 (0.65–1.89)	1.05 (0.44–2.49)
5 +	1.27 (1.06–1.53)	1.13 (0.93–1.38)	1.41 (0.87–2.29)	1.84 (0.89–3.79)
Unknown	1.84 (0.23–14.62)	1.59 (0.36–6.99)	ISS	ISS
**Ethnicity**				
White	1.26 (0.61–2.62)	1.54 (0.67–3.52)	0.64 (0.16–2.62)	0.44 (0.06–3.26)
Mixed ethnic groups	1.24 (0.3–5.04)	1.75 (0.41–7.47)	ISS	ISS
Black	Ref	Ref	Ref	Ref
Asian	1.63 (0.75–3.55)	2 (0.83–4.84)	0.28 (0.04–1.99)	1.26 (0.14–11.01)
Others	1.47 (0.66–3.24)	1.63 (0.66–4)	0.85 (0.17–4.23)	0.49 (0.04–5.58)
Missing	1.14 (0.55–2.38)	1.45 (0.63–3.33)	0.47 (0.11–1.96)	0.36 (0.05–2.76)
**BMI, kg/m**^**2**^				
Underweight (< 18.5)	1.36 (1.03–1.81)	1.31 (0.97–1.76)	1.2 (0.51–2.85)	1.52 (0.45–5.09)
Normal weight (18.5–24.9)	Ref	Ref	Ref	Ref
Overweight (25–29.9)	0.98 (0.86–1.12)	0.93 (0.81–1.06)	1.26 (0.88–1.81)	2.09 (1.23–3.57)
Obese(> 30)	1.11 (0.98–1.26)	1.05 (0.92–1.2)	1.34 (0.94–1.93)	1.67 (0.93–2.98)
Missing	1.31 (1.15–1.49)	1.28 (1.11–1.47)	1.23 (0.84–1.82)	1.52 (0.82–2.81)
**Smoking history**				
Never smoked	Ref	Ref	Ref	Ref
Current smoker	1.44 (1.28–1.61)	1.4 (1.24–1.58)	1.67 (1.22–2.29)	2.14 (1.33–3.43)
Former smoker	0.85 (0.73–0.99)	0.89 (0.75–1.04)	0.93 (0.6–1.45)	0.79 (0.38–1.67)
Not known	1.4 (1.18–1.65)	1.31 (1.1–1.56)	1.52 (0.95–2.45)	1.94 (0.97–3.89)

*ISS* Insufficient Sample Size

Multimorbidity at the time of conception was associated with higher risk of neonatal admission (aOR = 1.35, 95% CI: 1.19–1.53). However, no significant association was found with stillbirth (aOR = 0.67, 95% CI: 0.39–1.16) or neonatal death (aOR = 1.28, 95% CI: 0.64–2.59) (Additional file 7: Table 5).

## Discussion

### Main findings

In this retrospective study, we found that multimorbidity at the time of conception was present in one in six women and was associated with an overall increased risk for PTB compared to women with no multimorbidity. We also observed an increased risk of neonatal admissions.

### Comparison with the literature

Our findings suggest that multimorbidity is prevalent among pregnant women and is associated with adverse perinatal outcomes. This aligns with previous literature that has reported associations between individual or combination of chronic conditions and diverse adverse obstetric and neonatal outcomes [9, 23–25]. A significant burden of multimorbidity has been previously reported in women of childbearing age [3] and pregnant women [4, 5, 26, 27]. Our results show a lower prevalence than Lee et al. reported, where our data from 2018 were included. This is likely related to the fact that our measurement of multimorbidity is based on hospital records which may underestimate the prevalence of multimorbidity compared with studies using primary care data.

Recently, Naksini K et al. published the first paper investigating the association between maternal multimorbidity and perinatal outcomes [10]. Their study reported a lower prevalence of multimorbidity (6.3%) compared to ours and to previous studies [4, 28], which can be attributed to differences in the definition of multimorbidity. They included fewer conditions than our study and utilised self-administered questionnaires and medical record transcripts during the first trimester of pregnancy, which may lead to underreporting certain conditions. It is important to consider that their study was conducted in Japan, and the prevalence of chronic conditions in this population may differ significantly from that in the UK. Furthermore, they found a lower prevalence of preterm birth (4.6%) compared to our study but a significant association with maternal multimorbidity similar to our findings (aOR = 1.50, 95% CI: 1.33–1.69). In a small prospective cohort study of pregnant women (*n* = 1619) conducted more than 15 years ago, Hass J.S et al. [29] found that after adjusting for sociodemographic characteristics, pre-pregnancy risk factors, and pregnancy risk factors, women who reported poor physical function during the month before conception had nearly a twofold increased likelihood of experiencing a PTB (aOR = 1.97, 95% CI: 1.18–3.30) as women with better physical function. They concluded that a broader focus on women's health before pregnancy may reduce PTB rates. Our findings are consistent; women with pre-pregnancy multimorbidity have a higher risk of PTB.

Two other studies [8, 26] found a dose–response relationship between the number of identified conditions and the rate of severe maternal morbidity. As the number of identified conditions increased, the rate of severe maternal morbidity, defined by a combination of adverse events at the time of delivery, also increased. These findings align with this study, and they used a similar methodology (hospital records and ICD10 codes to diagnose both maternal morbidity and chronic conditions); however, there are relevant differences in how our results can be interpreted and the potential impact in clinical practice. In all these previous studies, assessment of exposure (chronic conditions) and outcomes was only performed at the time of birth, so the timing of diagnosis could not be ascertained. Conversely, our estimation of multimorbidity at the time of conception suggests that women at higher risk can be identified at the start of their pregnancy [9].

### Clinical and research implications

It is well known that PTB causes higher mortality and long-term morbidity for the child, but its causes are not yet fully understood. Preterm labour is considered a syndrome initiated by multiple mechanisms, including infection or inflammation, uteroplacental ischaemia or haemorrhage, uterine overdistension, stress, and other immunologically mediated processes [30]. However, a precise mechanism cannot be established in most cases.

Women entering pregnancy with multimorbidity have more than a 64% increased risk of PTB. These findings warrant further research into potential causal pathways. Given the increased risk of other adverse outcomes associated with PTB, there is an urgent need for a more holistic approach to the care provided to women with multimorbidity before and during pregnancy. It is important to highlight that multimorbidity increases the risk of PTB across all ages and socioeconomic levels. This suggests a benefit of addressing or improving the care of women with multimorbidity in the pre-conception period. Maternal multimorbidity should be considered as an independent risk factor of poorer perinatal outcomes beyond the high risk pregnancy consideration based on individual conditions. To be able to define a clear population at risk is the first step in developing specific interventions. Our results suggest that pregnant women with multiple long-term conditions should be prioritised to explore possible interventions. These results also indicate that interventions and preventive strategies should start before pregnancy and support the existing calls from multiple professional bodies and National reviews for pre-pregnancy counselling to be readily available for women with pre-existing medical and mental health morbidity [31, 32].

### Strengths

This study utilised electronic health records, which provided a rich source of data and it avoided misclassification bias associated with self-reported surveys. Data from Information Services Division, NHS Scotland [33] show that SMR02 records contain almost 99% of all births registered in Scotland between 2014–18. This suggests that the cohort within this study contains the vast majority of all births over the period, and the risk of potential selection bias and underrepresentation of subgroups is low. We have used a comprehensive definition of multimorbidity that includes 79 conditions, prioritised by a multidisciplinary group, including patient representatives, which has been previously published. The use of diagnosis codes allows these findings to be validated in other contexts and ensures reproducibility.

### Limitations

As with all research that uses routine health records, it is subject to the availability and quality of data entry and confounding. Our cohort had a minimum 5-year look back period to allow for ascertainment of multimorbidity, and it may be that a longer period may show higher levels. The high proportion of missing data in some variables introduces a limitation of our study. Given the inherent complexity of our data and the potential biases introduced by imputation, we believe that multiple imputation was unsuitable for addressing our non-random missing data. Instead, as there is no universally perfect approach to handling missing data, especially when using EHRs, we opted to reflect the missingness as it occurred in our dataset and acknowledge this as a limitation. A complete case analysis is presented in the supplementary information for comparison (Additional file 8: Table 6). We have been unable to differentiate between the different types of PTB (spontaneous versus medically indicated), which limits our ability to make assumptions on how each individual condition contributed or to theorise regarding causality [34]. Previous studies have reported racial and ethnic disparities in both the incidence of severe maternal morbidity and the prevalence of multimorbidity [35]. Our population was mainly of white ethnicity, and we had 20% missing data in this variable. Larger and more diverse populations may be better positioned to confirm this hypothesis.

Another possible limitation to comparing our results with other studies is that we have excluded any birth recorded as less than 24 weeks of gestational age. We had only identified 34 records that could have been considered live births between 22 and 24 weeks of gestation, so this is unlikely to have changed our findings. Finally, as we used mainly hospital data to estimate multimorbidity, it is possible that we have captured more severe cases, and our results may differ from studies that used primary care data.

## Conclusions

Maternal multimorbidity at the time of conception was associated with an increased risk of PTB and neonatal admission. Multimorbidity presents a significant health burden to women and their offspring. Our findings suggest that routine and comprehensive assessments for women with multimorbidity before and throughout pregnancy could optimise care and ultimately improve outcomes for them and their offspring.

### Supplementary Information

**Additional file 1.** Detailed information on the datasets and data source used for this analysis.

**Additional file 2.** Cohort selection and data quality checks.

**Additional file 3.** Phenome definition of health conditions and ICD 10 codes used for identifying long-term conditions.

**Additional file 4.** Outcome and variable definition and details on how variables are recorded in the original data source.

**Additional file 5.** Reporting of studies Conducted using Observational Routinely-collected Data: The RECORD Statement Checklist.

**Additional file 6: Table 4.** Association between maternal multimorbidity and preterm birth: Stratified regression model using Mantel-Haenszel.

**Additional file 7: Table 5.** Association between maternal multimorbidity and other perinatal outcomes.

**Additional file 8: Table 6.** Multivariate adjusted association between maternal multimorbidity and preterm birth complete case analysis.

## Data Availability

The data that support the findings of this study are available from the HIC at the University of Dundee, but restrictions apply to the availability of these data, which were used under license for the current study, and so are not publicly available. All code used in this study is publicly available at https://github.com/mumpredict.

## Abbreviations

PTB: Preterm birth
ORs: Odds Ratios
EHRs: Electronic Health Records
SMR: Scottish Morbidity Records
SMR02: Scottish Maternity Records
BMI: Body mass index
NHS: National Health Service
LMP: Last menstrual period
SIMD: Scottish Index of Multiple Deprivation
UK: United Kingdom
