# Supplementary material for: Maternal multimorbidity and preterm birth in Scotland: an observational record-linkage study
Source: BMC Med. 2023 Sep 12;21:352. doi: 10.1186/s12916-023-03058-4 (PMC10496247; doi:10.1186/s12916-023-03058-4)
Supplement: Supplementary file 1 — Additional file 1. Detailed information on the datasets and data source used for this analysis. [file 12916_2023_3058_MOESM1_ESM.docx]

# **Additional file 1: Data source**

Secondary (hospital) healthcare data for individual patients is collected as a series of Scottish Morbidity Records (SMR) provided for this project by the Health Informatic Centre from the University of Dundee from two regions, Tayside and Fife. <https://www.dundee.ac.uk/hic/data-service/dataset-inventory> .

SMR databases are episode-based records relating to all inpatients and day cases discharged from wards in Scottish hospitals. The record type denotes the general type of healthcare received during an episode and/or the nature or status of the patient. The data sets used for this project are:

1. **Maternity and Neonatal Data-base (SMR02).** Used to identify the study population. SMR02 is linked separately and contains obstetric discharge records for all mothers delivering in Scotland since 1975 as well as baby records relating to mothers’ offspring. Records relating to the same mother and all of her offspring are permanently linked together in this data set. SMR02 is submitted by maternity hospitals to the Information Service Division (ISD) which is part of National Services Scotland, part of NHS Scotland. It includes data on antenatal booking appointments,  mother’s age, deprivation, smoking history, alcohol and drug misuse, BMI (at antenatal booking); birth - induction, mode of delivery, outcome (live or still-birth); baby - gestation, and birth weight. A variety of data are available for different geographies and by deprivation quintile.
2. **Scottish Morbidity Database** contains records relating to non-obstetric hospital admissions (used to determine the presence of multimorbidity)
   1. SMR01-**General / Acute Inpatient and Day Case**
   2. SMR04- **Mental Health Inpatient and Day Case**
   3. A&E- **Accident and Emergency (A&E) Service**
   4. Demography and Death registrations
3. In addition, the Prescribing Information System (PIS) is the data source for all prescribing relating to all medicines and their costs that are prescribed and dispensed in the community in Scotland. PIS is available from the two included Scottish regions from 2009.

Linkage of different datasets and PIS is done using The Community Health Index (CHI) unique is a 10-character numeric identifier allocated to each patient on first registration with the system that is removed and not provided to the researchers.
