# Supplementary material for: Maternal multimorbidity and preterm birth in Scotland: an observational record-linkage study
Source: BMC Med. 2023 Sep 12;21:352. doi: 10.1186/s12916-023-03058-4 (PMC10496247; doi:10.1186/s12916-023-03058-4)
Supplement: Supplementary file 2 — Additional file 2. Cohort selection and data quality checks. [file 12916_2023_3058_MOESM2_ESM.docx]

# **Additional file 2: Cohort selection and data quality checks**

**Cohort selection**

● Index pregnancy is the pregnancy with a start date from 1st Jan 2014 to 31st December 2018.

● To estimate the pre-pregnancy multimorbidity when a woman has more than one pregnancy episode in that time frame, one pregnancy was selected at random and considered the index pregnancy for the prevalence analysis.

● To investigate the association of multimorbidity with PTB and other outcomes, all pregnancies in the 5-year period were included with pregnancies to the same women treated as related records.

● Age at pregnancy start date of 15 to 49 years old

● Women of these pregnancies need to have at least one year worth of data recorded preceding index pregnancy. Women resident in the area for at least one year, determined by the date of registering with a GP within the area.

**Pregnancy start date**

● Date of conception for index pregnancy based on last menstrual period (LMP) recorded at booking appointment or estimated using the gestational age in weeks at booking appointment or gestational age in weeks and delivery.

**Quality check:**

● Anonymized linked dataset within a Safe Haven environment was created and maintained using internationally accepted privacy-preserving protocols by Health Informatics Centre (HIC).

● Death date should be after index pregnancy start date

● Date the patient transferred out of the data-contributing health board should be after the index pregnancy start date

● Date of the last data collection for the data-contributing health board should be after the index pregnancy start date

● Patient’s registration date in the health board should be at least one year before the index pregnancy start date
