# Supplementary material for: Maternal multimorbidity and preterm birth in Scotland: an observational record-linkage study
Source: BMC Med. 2023 Sep 12;21:352. doi: 10.1186/s12916-023-03058-4 (PMC10496247; doi:10.1186/s12916-023-03058-4)
Supplement: Supplementary file 3 — Additional file 3. Phenome definition of health conditions and ICD 10 codes used for identifying long-term conditions. [file 12916_2023_3058_MOESM3_ESM.docx]

# Additional file 3

# Phenome definitions of health conditions

These diagnostic codes were based on existing literature and code list repositories and when not available, were generated by clinicians in the research team .

## Health conditions

- 79 health conditions that will count towards the multimorbidity status were identified in a workshop with women representatives and a multidisciplinary team.
- The multidisciplinary team consisted of generalists (GP) and specialists (obstetric, obstetric and foetal medicine, perinatal mental health, public health).
- These are health conditions that pre-existed at baseline prior to the conception of the index pregnancy.
- All historical medical records that were available in the datasets before the index pregnancy contributed to the identification of multimorbidity unless specified otherwise. The absence of a health condition was considered when there were no relevant ICD-10 codes.
- Additional phenome definitions by community prescriptions and event dates were used to: (i) improve the detection of health conditions (e.g. identifying mental health conditions with relevant prescriptions), (ii) improve the accuracy of morbidity detection (e.g. regular topical steroids for atopic eczema), and (iii) limit childhood morbidities that may resolve in adulthood (e.g. asthma only considered if still present from age 11 years onwards (9).

## Transient conditions limited to childhood / episodic conditions

Certain childhood conditions are likely to resolve and not continue in adulthood, for example viral induced wheeze, cradle cap, atopic eczema. This is especially the case for atopic conditions and dermatological conditions. There are also conditions that are less likely to occur in very early childhood, such as mental health conditions and chronic headache (except neurodevelopmental disorder and migraine). We anticipate the documentation of these conditions will be high or over-diagnosed in primary care records and therefore have agreed on additional phenome definitions with our clinical colleagues.

We would be less interested in conditions that were transient in childhood and subsequently never active again in a woman’s lifetime. To define disease in adulthood, we have taken into account that age 16 is the age for lawful consent and where a patient may transition to adult health care services. However, using a definition of disease occurring in adulthood (aged >16) would discount recent medical history for the youngest pregnant women in the study cohort. Therefore, we have chosen aged 11 (5 years before 16 years old) as the cut off for these conditions.

The following health conditions will only be considered if the latest related health record is when the woman is aged 11 and above:

- all mental health conditions (excluding neurodevelopmental disorders),

- atopic eczema,

- other skin conditions (seborrheic dermatitis, rosacea, hidradenitis suppurativa, lichen planus),

- allergic rhino-conjunctivitis,

- asthma

- chronic headache (cluster headache, tension headache, chronic type headache)

## Analysis of mental health conditions

*Common mental health disorders (CMHD)*

Depression and anxiety diagnoses were treated as separate morbidities. Mixed depression and anxiety is the most common mental health disorder (10), but as the maternal outcome may differ for these conditions (11), we analysed them separately where possible.

Medications used to manage mental health conditions often are used in more than one health condition. For instance, selective serotonin reuptake inhibitors are used in both depression and anxiety. Therefore, the prescription phenome for depression and anxiety were combined as common mental health disorders (CMHD) medications.

In the absence of a diagnosis code, it is not possible to determine whether the CMHD prescription was used for anxiety or depression. CMHD prescription phenome if present, will contribute as one morbidity, only when neither a depression nor anxiety diagnosis code is present.

*Severe mental illness (SMI)*

As the diagnosis codes and drugs used in bipolar disorder, schizophrenia and psychosis may overlap, and given that it is very uncommon for bipolar disorder and schizophrenia to co-occur, these conditions were combined as severe mental illness if a diagnosis or prescription code is present.

*Alcohol misuse/dependence, substance misuse/dependence*

Both these conditions were considered to be present if a diagnosis code or prescription code is present.

## Mental health conditions phenome definitions

| **Mental health conditions** | **Phenome definitions^a^** | **Prescription BNF chapters** | **Phenome by prescriptions** |
| --- | --- | --- | --- |
| Common mental health disorders (CMHD)   - *Depression* - *Anxiety*   *(includes phobia, panic disorder, post-traumatic stress disorder)* | - Diagnosis code - Prescription code: CMHD prescriptions - Improving access to psychological therapy (IAPT) program | **CMHD prescriptions**   - 4.3: Antidepressant drugs (excluding amitriptyline) - 4.1.2 Anxiolytics - Propranolol 10mg, 40mg | 4 CMHD prescriptions within 12 months *AND* no lifetime SMI prescriptions |
| Severe mental illness (SMI)   - *Bipolar disorder* - *Schizophrenia* - *Affective psychosis* - *Non affective psychosis* | - Diagnosis code - Prescription code: SMI prescriptions - Improving access to IAPT program | **SMI prescriptions**  **Group A:**  4.3: Antidepressant drugs (excluding amitriptyline)  4.1.2 Anxiolytics  **Group B:**  4.2.1: Antipsychotic drugs (excluding prochlorperazine)  4.2.2: Antipsychotic depot injections  **Group C:**  4.2.3: Drugs used for mania and hypomania (lithium, asenapine) | 4 group A prescriptions within 12 months AND any lifetime group B/C prescriptions  *OR*  4 group B prescriptions within 12 months  *OR*  4 group C prescriptions within 12 months |
| Alcohol misuse/dependence | - Diagnosis code - Prescription code | 4.10.1 Alcohol dependence (acamprosate, disulfiram, nalmefene) | 4 prescriptions within 12 months |
| Substance misuse/dependence | - Diagnosis code - Prescription code | Methadone | 4 prescriptions within 12 months |
| Eating disorder | - Diagnosis code | - | - |
| Neurodevelopmental disorder   - *Attention deficit hyperactivity disorder* - *Autism* - *Learning difficulties* | - Diagnosis code - Prescription code (SMR only) | Methylphenidate | 4 prescriptions within 12 months |
| Other mental health conditions   - *Obsessive compulsive disorder* - *Personality disorder* - *Dissociative disorder* - *Self-harm (including suicide)* | - Diagnosis code | - | - |

^a^ Latest diagnosis code and prescription codes at aged 11 or above, except for neurodevelopmental disorder

BNF: British National Formulary

## Physical health conditions phenome definitions

| **Health conditions** | **SMR** | **British National Formulary chapters for phenome by prescriptions** |
| --- | --- | --- |
| **Cancers** |  |  |
| All cancers | Diagnosis code |  |
|  |  |  |
| **Cardiovascular disease** |  |  |
| Hypertension | Diagnostic OR prescription code.  4 prescriptions in 12months anytime. | 2.2.1: Thiazides and related diuretics; 2.6.2: Calcium-channel blockers; 2.5.5: Renin-angiotensin system drugs; 2.5.4: Alpha-adrenoceptor blocking drugs |
| Ischemic heart disease & myocardial infarction | Diagnosis code |  |
| Heart failure | Diagnosis code |  |
| Stroke | Diagnosis code |  |
| Atrial fibrillation | Diagnosis code |  |
| Congenital heart disease | Diagnosis code |  |
| Valvular heart disease | Diagnosis code |  |
| Cardiomyopathy | Diagnosis code |  |
|  |  |  |
| **Dermatology** |  |  |
| Atopic eczema | Topical steroid prescription OR diagnostic code aged 11+.  4 prescriptions in 12months. | 13.4: Topical corticosteroids |
| Psoriasis | 4 prescriptions in 12months | 13.5.2: Preparations for psoriasis |
| Autoimmune skin disease  (vitiligo, alopecia areata) | Not available |  |
| Other skin conditions  (Seborrheic dermatitis, Rosacea, Hidradenitis suppurativa, Lichen planus) | Diagnosis code |  |
|  |  |  |
| **Ear, nose throat** |  |  |
| Allergic rhinoconjunctivitis | 2 prescriptions in 6 months aged 11+ | 12.2.1 Nasal allergy topical antihistamines, cromoglicate, topical corticosteroids |
|  |  |  |
| Profound deafness | Diagnosis code |  |
|  |  |  |
| **Eye** |  |  |
| Severe blindness | Diagnosis code |  |
| Inflammatory eye disease  (scleritis, episcleritis, uveitis) | Diagnosis code |  |
| Cataract | Diagnosis code |  |
| Diabetic eye disease  (retinopathy, maculopathy) | Diagnosis code |  |
| Retinal detachment | Diagnosis code |  |
|  |  |  |
| **Gastroenterology** |  |  |
| Irritable bowel disease | Diagnosis code OR prescription 4 prescription in 12 months | 1.2: Antispasmodics and other drugs altering gut motility |
| Inflammatory bowel disease  (ulcerative colitis, Crohn's disease) | Diagnosis code |  |
|  |  |  |
| Coeliac disease | Diagnosis code |  |
| Chronic liver disease  (chronic hepatitis B & C, alcoholic liver disease, autoimmune liver disease, cirrhosis) | Diagnosis code |  |
| Non-alcoholic fatty liver disease | Diagnosis code |  |
| Peptic ulcer disease | Diagnosis code OR prescription 4 prescription in 12 months | 1.3.1: H2-receptor antagonists, 1.3.5: Proton pump inhibitors |
| Cholelithiasis | Diagnosis code |  |
|  |  |  |
| **Gynaecology** |  |  |
| Polycystic ovarian syndrome | Diagnosis code |  |
| Endometriosis | Diagnosis code |  |
| Leiomyoma (fibroids) | Diagnosis code |  |
| Female infertility | Diagnosis code |  |
|  |  |  |
| **Haematology** |  |  |
| Venous thromboembolism (VTE)  (deep vein thrombosis, pulmonary embolism, other VTE) | Diagnosis code |  |
| Primary thrombocytopenia | Diagnosis code |  |
| Haemophilia | Diagnosis code |  |
| Pernicious anaemia | Diagnosis code |  |
| Sickle cell anaemia | Diagnosis code |  |
|  |  |  |
| **Rheumatology** |  |  |
| Systemic lupus erythematosus | Diagnosis code |  |
|  |  |  |
| Spondylarthritis  (psoriatic arthritis, ankylosing spondylitis) | Diagnosis code |  |
|  |  |  |
| Inflammatory arthritis  (rheumatoid arthritis, Sjogren’s syndrome, Raynaud's syndrome, systemic sclerosis, primary systemic vasculitis) | Diagnosis code |  |
|  |  |  |
| Ehlers’s Danlos Syndrome (EDS): Type 3 (Hypermobile EDS) | Diagnosis code |  |
|  |  |  |
| **Orthopaedic** |  |  |
| Scoliosis | Diagnosis code |  |
| Vertebral disorder  (intervertebral disc disorder, spondylosis, spondylolisthesis, collapsed vertebrae, spinal stenosis) | Diagnosis code |  |
| Chronic back pain | Diagnosis code |  |
| Osteoporosis | Diagnosis code |  |
| Osteoarthritis | Diagnosis code |  |
|  |  |  |
| **Neurology** |  |  |
| Migraine | 4 prescriptions in 12 months | 4.7.4: Antimigraine drugs |
| Other chronic headaches  (cluster headache, tension headache) | Not available |  |
|  |  |  |
|  |  |  |
| Epilepsy | Diagnostic code OR Prescription: antiepileptics specific to epilepsy | 4.8.1: Control of epilepsy (exclude Gabapentin [0408010G0], Pregabalin [0408010AE] and Topiramate [040801050]) |
| Multiple sclerosis | Diagnosis code |  |
| Spina bifida | Diagnosis code |  |
| Idiopathic intracranial hypertension | Diagnosis code |  |
| Peripheral neuropathy | Diagnosis code |  |
| Somatoform disorder  (chronic fatigue syndrome / myalgic encephalomyelitis, fibromyalgia, chronic pain syndrome [chronic regional pain syndrome, myofascial pain syndrome]) | Diagnosis code |  |
|  |  |  |
| **Respiratory** |  |  |
| Asthma | 4 scripts in 12 months aged 11+ | 3.1.1: Adrenoceptor agonists OR 3.2: Corticosteroids |
| Chronic obstructive pulmonary disease | Not available |  |
| Obstructive sleep apnoea | Diagnosis code |  |
| Pulmonary fibrosis, interstitial lung disease | Diagnosis code |  |
| Pulmonary hypertension | Diagnosis code |  |
| Bronchiectasis | Diagnosis code |  |
| Cystic fibrosis | Diagnosis code |  |
| Sarcoidosis | Diagnosis code |  |
|  |  |  |
| **Renal** |  |  |
| Chronic kidney disease, dialysis | Diagnosis code |  |
| Urolithiasis | Diagnosis code |  |
| **Endocrine** |  |  |
| Type 1 diabetes mellitus | Diagnosis code OR prescription 4 prescription in 12 months | 6.1.1: Insulin |
| Type 2 diabetes mellitus | Diagnosis code OR prescription 4 prescription in 12 months AND no insulin | 6.1.2: Antidiabetic drugs (exclude Metformin Hydrochloride (0601022B0) due to its use for infertility |
| Hyperthyroidism | Diagnosis code OR prescription 4 prescription in 12 months | 6.2.2: Antithyroid drugs |
| Hypothyroidism | Diagnosis code OR prescription 4 prescription in 12 months | 6.2.1: Thyroid hormones |
| Pituitary disorder | Diagnosis code |  |
| Adrenal benign tumour | Diagnosis code |  |
| Hyperparathyroidism | Diagnosis code |  |
|  |  |  |
| **Other** |  |  |
| Human immunodeficiency viral (HIV) infection / AIDS | Diagnosis code |  |
| Turner’s syndrome | Diagnosis code |  |
| Marfan’s syndrome | Diagnosis code |  |
| Solid organ transplant | Diagnosis code |  |

## Phenome definitions to limit common transient / episodic conditions to the 12 months preceding index pregnancies

| **Health conditions** | **Phenome definitions** | **CPRD BNF Chapters** | **Scotland BNF Chapters** |
| --- | --- | --- | --- |
| Atopic eczema | Meets main criteria AND at least 1 prescription of topical steroid in the 12 months before index pregnancy | 13040000 Topical corticosteroids,  13040100 Topical corticosteroids with antimicrobials,  13040200 Mild topical corticosteroids,  13040300 Moderate topical corticosteroids,  13040400 Potent topical corticosteroids,  13040500 Very potent topical corticosteroids | 13.4: Topical corticosteroids |
| Psoriasis | Meets main criteria AND at least 1 prescription of (topical steroid OR topical psoriasis treatment) in the 12 months before index pregnancy | Topical steroids as above,  13050000 Preparations for eczema and psoriasis,  13050200 Preparations for psoriasis,  13050202 Topical preparations for psoriasis | 13.5.2: Preparations for psoriasis |
| Seborrheic dermatitis | Meets main criteria AND at least 1 prescription of (topical steroid OR topical antifungal treatment) in the 12 months before index pregnancy | Topical steroids as above,  13100200 Antifungal preparation (topical): included ketoconazole, miconazole, clotrimazole | - |
| Rosacea | Meets main criteria AND at least 1 prescription of (topical OR oral rosacea treatment) in the 12 months before index pregnancy | *Topical*  13060300 Topical preparations for rosacea, 13100102 Antibacterial preparations also used systemically (for skin conditions): included topical metronidazole,  13060101 Benzoyl peroxide and azelaic acid for acne: included azelaic acid.  *Oral*  03060201 Oral antibacterial for acne: included doxycycline, oxytetracycline, tetracycline, erythromycin,  13060000 acne and rosacea: doxycycline 40mg | *-* |
| Hidradenitis suppurativa | Meets main criteria AND at least 1 prescription of oral antibiotics for hidradenitis in the 12 months before index pregnancy | Lymecycline, metronidazole, clarithromycin, clindamycin, rifampicin,  13060201 Oral antibacterial for acne: included doxycycline, erythromycin. | - |
| Lichen planus | Meets main criteria and latest diagnosis code in the 12 months before index pregnancy | - | - |
| Allergic rhinoconjunctivitis | Meets main criteria by diagnosis code and at least 1 prescription for allergic rhinoconjunctivitis in the 12 months before index pregnancy | 12020100 Drugs used in nasal allergy  12020101 Corticosteroids used in nasal allergy (spray)  12020150 Antihistamines in nasal allergy (spray)  12020151 Cromoglicate in nasal allergy (spray) | 12.2.1 Drugs used in nasal allergy |
| Depression | Meets main criteria by diagnosis code and at least 1 prescription of antidepressant in the 12 months before index pregnancy | 04030100 Tricyclic and related antidepressants (excluded amitriptyline),  04030200 Monoamine oxidase inhibitors,  04030201 Reversible monoamine oxidase inhibitors,  04030300 Selective serotonin reuptake inhibitors,  04030400 Other antidepressants | 4.3 Antidepressant drug  4.1.1 Anxiolytics  propranolol (40 mg or 10 mg) |
| Anxiety | Meets main criteria by diagnosis code and at least 1 prescription of antidepressant/anxiolytics/propranolol in the 12 months before index pregnancy | Antidepressants as above,  04010000 Hypnotics and anxiolytics: included bromazepam  04010200 Anxiolytics,  04010201 Benzodiazepines  Propranolol 10mg, 40mg | 4.3 Antidepressant drug  4.1.1 Anxiolytics propranolol (40 mg or 10 mg) |
| Obsessive compulsive disorder | Meets main criteria and latest diagnosis code in the 12 months before index pregnancy | - | - |
| Self-harm | Meets main criteria and latest diagnosis code in the 12 months before index pregnancy | - | - |
| Migraine | Meets main criteria and at least 1 prescription of acute/prophylaxis migraine treatment in the 12 months before index pregnancy | 4070401 Acute Migraine, 4070402 Prophylaxis of Migraine | 4.7.4: Antimigraine drugs |
| Other chronic headaches | Meets main criteria and latest diagnosis code in the 12 months before index pregnancy | - | - |
| Asthma | Meets main criteria and at least 1 bronchodilator or steroid inhaler prescription in the 12 months before index pregnancy | 03010000 Bronchodilators, 03010101 Selective Beta 2 Agonists, 03020000 Corticosteroids (For Respiratory Conditions) | 3.1.1: Adrenoceptor agonists  3.2: Corticosteroids (respiratory) |

BNF: British National Formulary

# International Classification of Disease-version 10 (ICD-10) codes for health conditions

<https://github.com/mumpredict>
