# Supplementary material for: Maternal multimorbidity and preterm birth in Scotland: an observational record-linkage study
Source: BMC Med. 2023 Sep 12;21:352. doi: 10.1186/s12916-023-03058-4 (PMC10496247; doi:10.1186/s12916-023-03058-4)
Supplement: Supplementary file 4 — Additional file 4. Outcome and variable definition and details on how variables are recorded in the original data source. [file 12916_2023_3058_MOESM4_ESM.docx]

# **Additional file 4: Outcome and variable definitions**

# **Outcomes definitions**

| **Outcomes** | **Numerator** | **Denominator** |
| --- | --- | --- |
| Prevalence of multimorbidity  The exact definition is used for multimorbidity as the exposure variable for PTB. | Calculated at the estimated time of conception by the presence of 2 or more conditions from a predefined list of 79 conditions determined by a combination of codes from the International Classification of Disease 10th version (ICD-10) available in the Scottish Morbidity Records (SMR01, SMR04, A&E) and the Prescribing Information System women who had been a resident in the area for at least one year prior to the estimated conception date  For those women with more than one pregnancy in the period of interest, one pregnancy was selected at random to establish the prevalence of multimorbidity. However, we re-calculated prior to each pregnancy for the outcome analysis where more than one pregnancy from each woman had been included  The definition of multimorbidity and details in the ICD 10 codes have been previously published  doi.org/10.1186/s12884-022-04442-3  and is available here <https://github.com/mumpredict/Read-codes-and-ICD-10-codes> | Number of women having a recorded pregnancy in the 5-year period.  When a woman has more than one pregnancy episode in that time frame, one pregnancy was selected at random and considered the index pregnancy for the prevalence analysis. |
| Prevalence of complex multimorbidity | Presence of 4 or more long-term conditions using the same method as multimorbidity. | Number of women having a recorded pregnancy in the 5-year period. When a woman has more than one pregnancy episode in that time frame, one pregnancy was selected at random and considered the index pregnancy for the prevalence analysis. |
| Preterm birth | All live births with recorded gestational age between 24 weeks and less than 37. The variables *Estimated Gestation* or *Duration of Pregnancy* from SMR02 were used to establish this (see variable definitions) | Births recorded as livebirth with a recorded gestational age of 24 weeks or more |
| Moderate preterm (32 to <37 completed weeks of gestation) | All live births with recorded gestational age between 32 weeks and less than 37. | Births recorded as livebirth with a recorded gestational age of 24 weeks or more |
| Very preterm (28 to <32 weeks completed weeks of gestation | All live births with recorded or gestational age between 28 weeks and less than 32. | Births recorded as livebirth with a recorded gestational age of 24 weeks or more |
| Extremely preterm (24 to <28 weeks completed weeks of gestation) | All live births with recorded gestational age between 24 weeks and less than 28. | Births recorded as livebirth with a recorded gestational age of 24 weeks or more |
| Stillbirth | Births recorded as stillbirths | All births with recorded birth outcome (all births in cohort) |
| Neonatal deaths | Any recorded neonatal death, it will include   1. Livebirth dying within the first 6 days (early neonatal death). 2. Livebirth dying on or after the 7th completed day but before the 28th day (late neonatal death). 3. Livebirth dying on or after the 28th completed day but before the end of the first year of life (post-neonatal death). | All live births |
| Neonatal admission | Any recorded neonatal admission, regardless of the length. | All live births |
| Birth weight | As recorded in grams and categorise as follows: |  |
| Normal | >=2500 gr | All livebirths with known birthweight |
| Low Birth Weight | 1500 to 2499 gr | All livebirths with known birthweight |
| Very Low Birth Weight | 1000 to 1499 gr | All livebirths with known birthweight |
| Extremely Low Birth Weight | <1000gr | All livebirths with known birthweight |

# **Variable definition**

Variable definitions in our study are based on the available information in the SMR02- Maternity Inpatient and Day Case dataset, and we present them without making any changes to the original definitions or the names used for the variables please see <https://www.ndc.scot.nhs.uk/Data-Dictionary/SMR-Datasets/SMR02-Maternity-Inpatient-and-Day-Case/>

| **Variable** | **Data source** | **Variable Data Source Detail** |
| --- | --- | --- |
| **Age at conception (years)** | SMR02 | Recorded in completed years |
| **Previous live birth** | SMR02 | Number of previous live births prior to the current one |
| **Previous pregnancies** | SMR02 | The total number of pregnancies experienced by the woman prior to the current one, including ectopic pregnancy and pregnancies ending in therapeutic or spontaneous abortion |
| **Ethnicity** | SMR02 | Grouped into White, Asian, Black, Mixed and Other according to <https://www.ndc.scot.nhs.uk/Dictionary-A-Z/Definitions/index.asp?Search=E&ID=243&Title=Ethnic%20Group> |
| **Deprivation (SIMD)** | SMR02 | Scottish Index of Multiple Deprivation (SIMD) combines seven different domains (aspects) of deprivation: income; employment; health; education, skills and training; geographic access to services; crime; and housing. Separated into quintiles |
| **BMI , kg/m^2^** | SMR02 | At booking appointment |
| **Smoking history** | SMR02 | Smoking history at booking |
| **Estimated date of conception** | SMR02 |  |
| **Gestational age at birth** | SMR02 | Two variables from the maternity records were used to establish this:   1. Estimated Gestation: The number of completed weeks of pregnancy, as judged by the clinician (doctor or midwife), usually on the basis of an ultrasound measurement. It can be recorded at any visit, including booking but MUST be completed for delivery and abortion episodes. 2. Duration of pregnancy: Recorded at birth   If not available or recorded as unknown, the case was not included. See flow chart |
| **Conception date** | SMR02 | This was established by the variable **Last Menstrual Period** available in the maternity records.  If this was not available, we estimated the conception date by considering the difference between   1. The birth date and gestational age in weeks recorded at that time 2. The gestational age recorded at booking when the pregnancy had not ended. |
| **Mode of delivery** | SMR02 | **As recorded in SMR02:**  0 Normal, spontaneous vertex vaginal delivery, occipito-anterior. 1 Cephalic vaginal delivery, with abnormal presentation of the head at delivery, without instruments, with or without manipulation 2 Low forceps, no rotation, forceps NOS (incl. Wrigleys). 5 Breech delivery, spontaneous, assisted or unspecified partial breech extraction. 6 Breech extraction, NOS. Version with breech extraction. 7 Elective (planned) caesarean section. 8 Emergency and unspecified caesarean section. 9 Other and unspecified method of delivery. A Mid cavity forceps, no rotation (incl. Haig Fergusson, Neville-Barnes etc). B Rotational forceps (incl Kiellands) C Ventouse, no rotation or unspecified D Ventouse with rotation E Other forceps delivery (includes ‘high-cavity’, high forceps)  **Recategorization of Mode of delivery as follow:**  0 and 1 as **Vaginal**  2, A,B,C,D,E as **Instrumental**  5, 6 as **Breech**  7 as **Elective C-section**  8 as **Emergency C-section** |
| **Neonatal admission** | SMR02 | As recorded : (NEONATAL INDICATOR BABY 1-3)  Length of admission (or non-admission) to a neonatal unit following delivery.  0 Not admitted  1 Admitted - for up to 48 hours  2 Admitted - for more than 48 hours  9 Not Known |
| SMR02 https://www.ndc.scot.nhs.uk/Data-Dictionary/SMR-Datasets/SMR02-Maternity-Inpatient-and-Day-Case/General-Definitions/  <https://www.ndc.scot.nhs.uk/Dictionary-A-Z/Definitions/index.asp?Search=E&ID=243&Title=Ethnic%20Group> | | |
