# Supplementary material for: Maternal multimorbidity and preterm birth in Scotland: an observational record-linkage study
Source: BMC Med. 2023 Sep 12;21:352. doi: 10.1186/s12916-023-03058-4 (PMC10496247; doi:10.1186/s12916-023-03058-4)
Supplement: Supplementary file 6 — Additional file 6: Table 4. Association between maternal multimorbidity and preterm birth: Stratified regression model using Mantel-Haenszel. [file 12916_2023_3058_MOESM6_ESM.docx]

# **Additional file 6: Table 4: Association between multimorbidity and preterm birth: Stratified regression model using Mantel-Haenszel**

| Table 5: Association between multimorbidity and preterm birth: Stratified regression model using Mantel-Haenszel by age and deprivation. | | | | |
| --- | --- | --- | --- | --- |
|  | **Preterm birth**  **(24 to < 37 w)**  Adjusted OR (95% CI) | **Moderate preterm**  **(32 to <37 w)**  Adjusted OR (95% CI) | **Very preterm**  **(28 to <32 w)**  Adjusted OR (95% CI) | **Extremely preterm**  **(24 to <28 w)**  Adjusted OR (95% CI) |
| **Age** |  | aOR(95% CI) | aOR(95% CI) | aOR(95% CI) |
| 15-19 | 1.16 (0.67-1.92) | 1.27 (0.72-2.15) | 0.57 (0.01-3.68) | 0.76 (0.02-5.17) |
| 20-24 | 1.79 (1.41-2.25) | 1.81 (1.41-2.31) | 1.67 (0.83-3.15) | 0.6 (0.12-2) |
| 25-29 | 1.7 (1.4-2.06) | 1.73 (1.41-2.12) | 1.37 (0.68-2.58) | 0.82 (0.21-2.4) |
| 30-34 | 2.04 (1.68-2.46) | 2.07 (1.69-2.53) | 1.57 (0.87-2.71) | 1.36 (0.45-3.45) |
| 35-39 | 2.29 (1.77-2.94) | 2.17 (1.65-2.83) | 2.36 (1.1-4.81) | 2.25 (0.6-7.25) |
| 40-44 | 1.76 (1.02-2.97) | 1.98 (1.14-3.35) | 0 (0-2.38) | 0 (0-7.18) |
| 45-49 | 0 (0-7.04) | 0 (0-5.32) |  |  |
| **Crude** | 1.85 (1.68-2.05) | 1.87 (1.69-2.08) | 1.53 (1.12-2.07) | 1.01 (0.58-1.67) |
| **MH combined** | 1.86 (1.68-2.05) | 1.88 (1.69-2.09) | 1.54 (1.15-2.07) | 1.04 (0.63-1.7) |
| **SIMD** |  |  |  |  |
|  |  |  |  |  |
| Most deprived | 1.59 (1.32-1.93) | 1.62 (1.31-1.98) | 1.22 (0.69-2.08) | 1.28 (0.5-2.96) |
| 2 | 1.67 (1.34-2.07) | 1.74 (1.38-2.18) | 1.06 (0.53-1.99) | 0.71 (0.18-2.08) |
| 3 | 1.64 (1.26-2.13) | 1.58 (1.19-2.09) | 2.18 (0.97-4.58) | 0.96 (0.18-3.36) |
| 4 | 1.99 (1.51-2.59) | 2.04 (1.53-2.69) | 1.81 (0.71-4.05) | 0.42 (0.01-2.72) |
| Less deprived | 2.22 (1.56-3.12) | 2.38 (1.66-3.36) | 0 (0-2.66) | 0.76 (0.02-5.39) |
| **Crude** | 1.78 (1.6-1.98) | 1.82 (1.62-2.03) | 1.43 (1.03-1.97) | 0.95 (0.53-1.6) |
| **MH combined** | 1.73 (1.56-1.92) | 1.77 (1.58-1.98) | 1.32 (0.97-1.81) | 0.9 (0.53-1.52) |
| SIMD: Scottish Index of Multiple Deprivation | | | | |
