# Supplementary material for: Maternal multimorbidity and preterm birth in Scotland: an observational record-linkage study
Source: BMC Med. 2023 Sep 12;21:352. doi: 10.1186/s12916-023-03058-4 (PMC10496247; doi:10.1186/s12916-023-03058-4)
Supplement: Supplementary file 7 — Additional file 7: Table 5. Association between maternal multimorbidity and other perinatal outcomes. [file 12916_2023_3058_MOESM7_ESM.docx]

# **Additional file 7: Table 5 Association between multimorbidity and other perinatal outcomes**

| **Table 4. Association between multimorbidity and perinatal outcomes** | | | |
| --- | --- | --- | --- |
|  | **Stillbirth** | **Neonatal admission** | **Neonatal deaths** |
|  | Adjusted OR (95% CI) | Adjusted OR (95% CI) | Adjusted OR (95% CI) |
| **Maternal multimorbidity** | 0.67(0.39-1.16) | 1.35(1.19-1.53) | 1.28(0.64-2.59) |
| No MM | Ref. | Ref. | Ref. |
| **Gestational age at birth** | | | |
| Preterm birth | NA | 16.29(14.6-18.17) | 22.02(11.7-41.46) |
| Term birth | NA | Ref. | Ref. |
|  | | | |
| **Age at conception, years** | | | |
| 15-19 | 0.9(0.36-2.29) | 0.79(0.62-1) | 0.72(0.15-3.38) |
| 20-24 | 1.6(0.92-2.78) | 0.92(0.79-1.08) | 1.06(0.43-2.66) |
| 25-29 | Ref. | Ref. | Ref. |
| 30-34 | 1.34(0.78-2.31) | 1.06(0.93-1.22)) | 0.92(0.4-2.1) |
| 35-39 | 1.43(0.73-2.77) | 1.13(0.95-1.33) | 1.25(0.5-3.15) |
| 40-44 | 1.46(0.43-4.96) | 1.47(1.11-1.98) | 1.26(0.26-6.12) |
| 45-49 | ISS | 0.68(0.15-3.12) | ISS |
| **SIMD** | | | |
| Most deprived 1 | 1.19(0.54-2.64) | 1.16(0.95-1.41) | 6.72(0.85-52.87) |
| 2 | 1.68(0.78-3.63) | 1.02(0.83-1.24) | 4.43(0.53-36.75) |
| 3 | 1.34(0.6-2.99) | 1.02(0.84-1.25) | 3.09(0.34-27.93) |
| 4 | 0.95(0.41-2.22) | 1.23(1.02-1.49) | 8.47(1.1-65.33) |
| Least deprived 5 | Ref. | Ref. | Ref. |
| Missing | 0.74(0.27-2) | 0.85(0.68-1.06) | 5.86(0.69-49.5) |
| **Previous pregnancies** | | | |
| 0 | Ref. | Ref. | Ref. |
| 1 | 0.66(0.38-1.14) | 0.82(0.72-0.94) | 1.57(0.69-3.56) |
| 2 | 0.78(0.42-1.45) | 0.82(0.7-0.96) | 1.15(0.42-3.15) |
| 3 | 1.51(0.81-2.82) | 0.83(0.69-1.01) | 1.02(0.3-3.44) |
| 4 | 1.49(0.68-3.26) | 1.04(0.82-1.32) | 2.17(0.68-6.96) |
| 5+ | 1.19(0.51-2.76) | 0.97(0.77-1.22) | 0.87(0.21-3.57) |
| Missing | 38.82(4.3-350.53) | 4.78(0.81-28.22) | 96.29(7.94-1167.65) |
| **Ethnicity** | | | |
| White | 0.48(0.06-3.5) | 1.59(0.66-3.84) | 1.33(0.56-3.17) |
| Mixed ethnic groups | ISS | 0.49(0.05-4.57) | ISS |
| Black | Ref. | Ref. | Ref. |
| Asian | 0.55(0.05-6.2) | 1.06(0.4-2.78) | 1.26(0.15-10.59) |
| Others | 0.75(0.07-7.43) | 1.67(0.64-4.33) | 5.61(1.52-20.67) |
| Missing | 0.34(0.04-2.64) | 1.13(0.46-2.74) | ISS |
| **BMI, kg/m2** | | | |
| Underweight (<18.5) | 0.46(0.06-3.39) | 1.08(0.75-1.57) | ISS |
| Normal weight (18.5-24.9) | Ref. | Ref. | Ref. |
| Overweight (25-29.9) | 1.55(0.91-2.62) | 1.16(0.99-1.34) | 1.77(0.82-3.83) |
| Obese(>30) | 1.27(0.71-2.25) | 1.3(1.12-1.51) | 0.4(0.11-1.41) |
| Missing | 1.24(0.68-2.26) | 1.73(1.49-2.01) | 1.55(0.67-3.58) |
| **Smoking history** | | | |
| Never smoked | Ref. | Ref. | Ref. |
| Current smoker | 2.7(1.65-4.42) | 1.12(0.98-1.29) | 0.79(0.36-1.72) |
| Former smoker | 1.78(0.97-3.25) | 0.93(0.78-1.11) | 0.57(0.17-1.91) |
| Missing | 2.9(1.44-5.82) | 1.16(0.96-1.39) | 0.59(0.18-1.88) |
| Adjusted for maternal age, socioeconomic status, ethnicity, number of previous pregnancies, BMI and smoking history. And gestational age. SIMD: Scottish Index of Multiple Deprivation. BMI: body mass index ISS Insufficient Sample Size SIMD: Scottish Index of Multiple Deprivation | | | |
