# Supplementary material for: Maternal multimorbidity and preterm birth in Scotland: an observational record-linkage study
Source: BMC Med. 2023 Sep 12;21:352. doi: 10.1186/s12916-023-03058-4 (PMC10496247; doi:10.1186/s12916-023-03058-4)
Supplement: Supplementary file 8 — Additional file 8: Table 6. Multivariate adjusted association between maternal multimorbidity and preterm birth complete case analysis. [file 12916_2023_3058_MOESM8_ESM.docx]

# **Additional file 8: Table 6 Multivariate adjusted association between maternal multimorbidity and preterm birth complete case analysis**

| **Table 6: Multivariate adjusted association between maternal multimorbidity and preterm birth complete case analysis** | | | | |
| --- | --- | --- | --- | --- |
|  | **Preterm birth**  **(24 to < 37 w)**  Adjusted OR (95% CI) | **Moderate preterm**  **(32 to <37 w)**  Adjusted OR (95% CI) | **Very preterm**  **(28 to <32 w)**  Adjusted OR (95% CI) | **Extremely preterm**  **(24 to <28 w)**  Adjusted OR (95% CI) |
| **Maternal multimorbidity** | 1.48 (1.28-1.72) | 1.53 (1.31-1.79) | 1.19 (0.79-1.8) | 0.88 (0.43-1.79) |
| No MM | Ref. | Ref. | Ref. | Ref. |
| **Age at conception, years** | | | | |
| 15-19 | 1.03 (0.81-1.31) | 0.99 (0.76-1.28) | 0.87 (0.42-1.77) | 2.93 (1.26-6.84) |
| 20-24 | 0.93 (0.78-1.1) | 0.9 (0.75-1.08) | 1.15 (0.72-1.84) | 1.42 (0.68-2.96) |
| 25-29 | 1(0-0) | Ref. | Ref. | Ref. |
| 30-34 | 0.97 (0.83-1.14) | 0.95 (0.8-1.13) | 1.12 (0.7-1.78) | 0.91 (0.42-1.97) |
| 35-39 | 1.3 (1.07-1.58) | 1.22 (0.99-1.5) | 1.52 (0.88-2.63) | 1.24 (0.5-3.07) |
| 40-44 | 1.36 (0.93-2) | 1.32 (0.88-1.97) | 1.08 (0.33-3.55) | 0.71 (0.08-6.32) |
| 45-49 | 0.53 (0.07-3.88) | 0.6 (0.08-4.37) | ISS | ISS |
| **SIMD** | | | | |
| Most deprived 1 | 1.28 (1.03-1.59) | 1.21 (0.97-1.52) | 2.91 (1.33-6.39) | 0.71 (0.27-1.86) |
| 2 | 1.24 (1-1.55) | 1.18 (0.94-1.48) | 2.76 (1.26-6.05) | 0.87 (0.34-2.25) |
| 3 | 1.12 (0.9-1.4) | 1.06 (0.84-1.33) | 2.2 (0.98-4.93) | 0.85 (0.32-2.26) |
| 4 | 1.04 (0.84-1.3) | 0.95 (0.76-1.2) | 2.24 (1-4.99) | 1.03 (0.4-2.67) |
| Least deprived 5 | Ref. | Ref. | Ref. | Ref. |
| **Previous pregnancies** | | | | |
| 0 | Ref. | Ref. | Ref. | Ref. |
| 1 | 0.72 (0.62-0.83) | 0.78 (0.67-0.92) | 0.45 (0.28-0.71) | 0.56 (0.3-1.06) |
| 2 | 0.78 (0.65-0.93) | 0.81 (0.67-0.98) | 0.59 (0.35-0.99) | 0.54 (0.24-1.25) |
| 3 | 0.85 (0.68-1.07) | 0.82 (0.64-1.05) | 1.11 (0.65-1.9) | 0.41 (0.11-1.46) |
| 4 | 1.14 (0.86-1.5) | 1.18 (0.88-1.58) | 0.9 (0.42-1.96) | 1.46 (0.52-4.15) |
| 5+ | 1.19 (0.9-1.58) | 1.02 (0.74-1.39) | 1.53 (0.8-2.92) | 1.86 (0.67-5.18) |
| **Ethnicity** | | | | |
| White | 1.11 (0.44-2.79) | 1.7 (0.52-5.52) | 0.35 (0.08-1.46) | 0.22 (0.03-1.65) |
| Mixed ethnic groups | 1.26 (0.23-7.03) | 2.27 (0.35-14.56) | ISS | ISS |
| Black | Ref. | Ref. | Ref. | Ref. |
| Asian | 1.27 (0.47-3.43) | 1.98 (0.57-6.82) | 0.12 (0.01-1.36) | 0.42 (0.04-4.99) |
| Others | 1.13 (0.37-3.48) | 1.79 (0.46-6.93) | ISS | 0.58 (0.04-9.52) |
| **BMI , kg/m^2^** | | | | |
| Underweight (<18.5) | 1.4 (1.01-1.93) | 1.45 (1.04-2.03) | 0.69 (0.21-2.3) | 1.16 (0.27-5.05) |
| Normal weight (18.5-24.9) | Ref. | Ref. | Ref. | Ref. |
| Overweight (25-29.9) | 1.04 (0.9-1.21) | 1 (0.86-1.17) | 1.17 (0.78-1.77) | 2.09 (1.12-3.87) |
| Obese(>30) | 1.1 (0.95-1.28) | 1.03 (0.88-1.2) | 1.29 (0.86-1.93) | 1.68 (0.86-3.28) |
| **Smoking history** | | | | |
| Never smoked | Ref. | Ref. | Ref. | Ref. |
| Current smoker | 1.39 (1.2-1.62) | 1.31 (1.12-1.54) | 1.72 (1.16-2.55) | 2.55 (1.39-4.66) |
| Former smoker | 0.78 (0.64-0.96) | 0.82 (0.67-1.02) | 0.88 (0.5-1.54) | 0.97 (0.4-2.37) |
| Adjusted for maternal age, socioeconomic status, ethnicity, number of previous pregnancies, BMI and smoking history. SIMD: Scottish Index of Multiple Deprivation. BMI: body mass index ISS Insufficient Sample Size | | | | |
